# Clinical Features and Prognostic Factors of 245 Portuguese Patients Hospitalized With COVID-19

**DOI:** 10.7759/cureus.13687

**Published:** 2021-03-04

**Authors:** Pedro Salvador, Pedro Oliveira, Tiago Costa, Mariana Fidalgo, Raul Neto, Maria Leonor Silva, Cristóvão Figueiredo, Vera Afreixo, Tiago Gregório, Luís Malheiro

**Affiliations:** 1 Internal Medicine Department, Centro Hospitalar Vila Nova De Gaia, Vila Nova de Gaia, PRT; 2 Internal Medicine Department, Centro Hospitalar Vila Nova De Gaia, Vila Nova De Gaia, PRT; 3 Intensive Medicine Department, Centro Hospitalar Vila Nova de Gaia, Vila Nova de Gaia, PRT; 4 Infectious Diseases Department, Centro Hospitalar Vila Nova De Gaia, Vila Nova De Gaia, PRT; 5 Center for Research and Development in Mathematics and Applications, Department of Mathematics, University of Aveiro, Aveiro, PRT

**Keywords:** coronavirus, covid-19, mortality, artificial respiration, hospitalization, europe

## Abstract

Introduction

Since the declaration of the severe acute respiratory syndrome coronavirus 2 (SARS-CoV-2) pandemic in March 2020, Portugal was considered a role model with regards to the first COVID-19 wave. However, a third wave started in 2021 started, turning the country into the worst in the world regarding new infections and death rate per capita in the last weeks of January 2021. No significant data regarding the country’s first wave of hospitalized patients have been published. Those data may help understand the differences over time regarding patients and the clinical approach to them. Herein, we present data of COVID-19 patients hospitalized at the main tertiary hospital of the second-most affected county at the time and identify risk factors associated with disease progression and outcomes.

Materials and methods

We performed a prospective observational study of patients admitted with COVID-19 to a central hospital between March 20 and June 1, 2020. The primary endpoint of this study was 30-day mortality or the need for ventilatory support and the secondary outcomes were both outcomes individually.

Results

245 patients were included, with a median age of 79 years, 52% males. Hypertension (n = 172) and dyslipidemia (n = 114) were the most frequent comorbidities. Half of the patients (n = 121) were treated with hydroxychloroquine. The primary outcome occurred in 114 patients; mortality at 30 days was 35%. Age (OR 1.05; 1.02-1.07) and active cancer (OR 3.89; 1.43-10.57) were associated with the primary outcome, with dyslipidemia being protective (OR 0.46; 0.25-0.80). Treatment with hydroxychloroquine or lopinavir/ritonavir was not associated with the main outcome. Patients who had been symptomatic for more than 7 days had lower mortality (OR 0.23; 0.09-0.63).

Discussion

In the present study, age and cancer were associated with higher mortality, as noted in prior articles. The population had a higher median age than reported in previous studies, which may explain the increased mortality. The protective association of dyslipidemia was not previously described. This association was not related to statin intake.

Conclusion

The reported high mortality of COVID-19 is rarely seen in other infectious diseases. Our elderly population probably reflects more reliably the incidence of COVID-19 in European countries with constricted age pyramids.

## Introduction

In December 2019, with the identification of a novel coronavirus - the severe acute respiratory syndrome coronavirus 2 (SARS-CoV-2) - in the city of Wuhan, China, the epidemiology of the world changed dramatically. As the new disease, named coronavirus 2019 (COVID-19) by the World Health Organization (WHO), rapidly spread across the globe, a worldwide pandemic was declared in March 2020. Since then, millions of people have been infected and died, making it the worst pandemic crisis since the Spanish flu. In Portugal, the first confirmed case was diagnosed on February 26, 2020, in a symptomatic man returning from Italy. In the first months of the pandemic, the northern part of the country became the main epicenter and, by the beginning of June 2020, more than 30,000 patients had been diagnosed in Portugal, with approximately 1400 deaths [1]. Retrospectively, those numbers seem few, as compared with the third wave that made the country the worst in the world regarding new infections and death rate per capitain the last weeks of January 2021 [2]. Among various reasons that might have led to this (including the newly identified SARS-CoV-2 variants), it is important to understand if the patients' characteristics and approach were different at the time.

Severe COVID-19 is a complex disease whose various clinical manifestations include respiratory failure with acute respiratory distress syndrome (ARDS) and need for mechanical ventilation; cardiac and cardiovascular complications with arrhythmias, myocarditis, and shock; thromboembolic complications with pulmonary embolism and stroke; and an inflammatory condition similar to the cytokine release syndrome [3-4]. The reasons behind some patients progressing to severe disease with others being asymptomatic or mildly ill have not been totally clarified.

The aim of this study is to describe the clinical characteristics of Portuguese patients with confirmed SARS-CoV-2 infection admitted to a tertiary hospital and to identify the risk factors associated with disease progression and outcomes.

## Materials and methods

We performed a prospective observational study of all adult patients admitted to Centro Hospitalar de Vila Nova de Gaia/Espinho, Portugal, due to COVID-19 between March 20 and June 1, 2020. All patients had confirmed SARS-CoV-2 infection diagnosed by reverse transcription-polymerase chain reaction (RT-PCR) in a respiratory tract sample. Patients were excluded if they were admitted for reasons other than COVID-19.

Institutional Ethics committee approval was obtained for this study. Data were collected by reviewing medical records and stored according to ethical concerns and data protection laws. The following parameters regarding clinical information prior to hospitalization were evaluated on admission: sex, age, length of symptoms before admission, arterial hypertension, diabetes mellitus, obesity, dyslipidemia, active cancer, chronic pulmonary disease (including asthma and COPD), chronic heart disease (heart failure, coronary artery disease, and/or cardiomyopathy), chronic kidney disease (CKD), atrial fibrillation, immunosuppression, and previous treatment with angiotensin-converting enzyme inhibitors (ACEI), angiotensin receptor blockers (ARB), statins, and antiplatelet or antithrombotic therapy. During hospitalization, the worst COVID-19 clinical stage [5] and the use of hydroxychloroquine and/or lopinavir/ritonavir for disease treatment were also recorded. The main outcome of interest was the combined outcome of 30-day mortality or the need for ventilatory support (invasive or non-invasive). Secondary outcomes were both outcomes individually.

Data analysis was performed using the Statistical Package for the Social Sciences (SPSS) for Windows, Version 25.0. (Armonk, NY, USA: IBM Corp). Descriptive statistics were used to describe the study population characteristics at baseline. Discrete variables were presented as absolute frequencies and percentages. Continuous variables were presented as mean ± standard deviation or median (interquartile range) according to their distribution. Categorical variables were compared using the chi-square test and continuous variables were compared using the students’ t-test and Mann-Whitney U test. The factors that presented a p-value <0.1 in the univariate analysis were included in a logistic regression model to determine the independent prognostic factors. A p-value of <0.05 was considered statistically significant. All p-values given are the result of two-sided tests.

## Results

During the inclusion period, a total of 271 admissions were considered, corresponding to 263 patients. Eighteen patients were excluded, as they were admitted for reasons other than COVID-19. The final study cohort included 245 patients, whose demographic data are presented in Table 1. The median age was 79 (66-86) years and the sex distribution was similar (52% male, n=127). Almost one-fourth had been symptomatic for over a week before admission (n=59). Arterial hypertension (70%, n=172) and dyslipidemia (47%, n=114) were the most common comorbidities. Statins were used by 43% (n=106) and ACEI/ARB by 42% (n=103). Almost half the patients were treated with hydroxychloroquine (49%, n=121) and 49 (20%) with lopinavir/ritonavir. The composite main outcome (mortality or ventilation at 30 days) occurred in 114 patients (47%). Mortality at 30 days was 35%. The age range in non-survivors varied between 59 and 101 years old.

**Table 1:** Demographic and descriptive data ACEI – angiotensin-converting enzyme inhibitors; ARB – angiotensin II receptor blockers; COVID-19 – coronavirus disease 2019; IQR – interquartile range

Variables	n	(IQR) or %
Age (years) – median	79	(66-86)
Sex (male)	127	52
Medical History		
Arterial hypertension	172	70
Chronic pulmonary disease	41	17
Obesity	45	18
Active cancer	25	10
Chronic kidney disease	47	19
Diabetes mellitus	78	32
Chronic heart disease	86	35
Immunosuppression	7	3
Dyslipidemia	114	47
Atrial fibrillation	41	17
Chronic medication		
ACEI/ARB	103	42
Statin	106	43
Antiplatelet	64	26
Anticoagulants	37	15
Symptoms for >7 days	59	24
Worst COVID-19 stage		
I	28	11
IIa	40	16
IIb	131	53
III	46	19
Drugs during hospitalization		
Hydroxychloroquine	121	49
Lopinavir/ritonavir	49	20
Length of stay (days) – median	8	(5-16)
Mortality or ventilation at 30 days	114	47
Mortality at 30 days	86	35
Ventilation at 30 days	44	18

Results from the univariate analysis evaluating the association between patient characteristics and simple and composite outcomes is described in Table 2. The results from the multivariable analysis evaluating the adjusted effect of patient characteristics in the outcomes can be found in Table 3.

**Table 2:** Composite and simple outcomes – univariate analyses ACEI – angiotensin-converting enzyme inhibitors; ARB – angiotensin II receptor blockers; CI – confidence interval; NS – not significant; OR – odds ratio

	30-day mortality/ventilation			30-day mortality			30-day ventilation		
Variables	OR	CI 95%	p	OR	CI 95%	p	OR	CI 95%	p
Age	1.04	1.02-1.06	<0.001	1.08	1.05-1.12	<0.001	0.97	0.95-0.99	0.006
Sex (male)	1.06	1.02-1.06	NS	1.11	0.66-1.88	NS	1.14	0.59-2-21	NS
Medical History									
Arterial hypertension	1.48	0.85-2.60	NS	2.00	1.10-3.77	0.027	0.89	0.45-1.84	NS
Chronic pulmonary disease	1.41	0.72-2.78	NS	0.73	0.34-1.48	NS	2.22	1.00-4.74	0.042
Obesity	0.51	0.25-0.99	0.052	0.54	0.25-1.10	NS	0.66	0.24-1.56	NS
Active cancer	2.69	1.15-6.85	0.027	3.15	1.36-7.57	0.008	1.16	0.37-3.07	NS
Chronic kidney disease	0.91	0.48-1.73	NS	1.48	0.77-2.83	NS	0.26	0.06-0.76	0.031
Diabetes mellitus	0.98	0.60-1.68	NS	1.05	0.60-1.84	NS	0.88	0.42-1.76	NS
Chronic heart disease	1.00	0.59-1.69	NS	1.57	0.91-2.71	NS	0.41	0.18-0.88	0.028
Immunosuppression	0.45	0.06-2.13	NS	0.30	0.02-1.80	NS	0.76	0.04-4.58	NS
Dyslipidemia	0.55	0.33-0.91	0.021	0.60	0.35-1.02	0.061	0.85	0.43-1.63	NS
Atrial fibrillation	1.11	0.57-2.19	NS	1.39	0.69-2.74	NS	0.59	0.19-1.48	NS
Chronic medication									
ACEI/ARB	0.82	0.49-1.36	NS	0.85	0.50-1.45	NS	0.84	0.43-1.63	NS
Statin	0.92	0.55-1.52	NS	0.73	0.43-1.25	NS	1.00	0.51-1.92	NS
Antiplatelet	1.21	0.68-2.14	NS	1.38	0.76-2.48	NS	0.58	0.24-1.26	NS
Anticoagulants	0.86	0.42-1.72	NS	1.15	0.55-2.35	NS	0.36	0.08-1.07	NS
Symptoms for >7 days	0.73	0.40-1.32	NS	0.30	0.13-0.60	0.001	2.70	1.34-5.39	0.005
Drugs during hospitalization									
Hydroxychloroquine	0.71	0.43-1.17	NS	0.34	0.20-0.59	<0.001	3.36	1.67-7.13	0.001
Lopinavir/ritonavir	0.60	0.31-1.14	NS	0.78	0.39-1-50	NS	0.72	0.28-1-64	NS

**Table 3:** Composite and simple outcomes – multivariate analyses CI – confidence interval; NS – not significant; OR – odds ratio

	30-day mortality/ventilation			30-day mortality			30-day ventilation		
Variables	OR	CI 95%	p	OR	CI 95%	p	OR	CI 95%	p
Age	1.05	1.02-1.07	<0.001	1.10	1.06-1.15	<0.001	0.97	0.94-1.01	NS
Arterial hypertension	-	-	-	1.71	0.73-3.98	NS	-	-	-
Chronic pulmonary disease	-	-	-	-	-	-	3.92	1.28-12.02	0.017
Obesity	0.63	0.29-1.35	NS	-	-	-	-	-	-
Active Cancer	3.89	1.43-10.57	0.008	6.94	2.27-21.21	0.001	-	-	-
Chronic kidney disease	-	-	-	-	-	-	0.32	0.07-1.02	NS
Chronic heart disease	-	-	-	-	-	-	0.12	0.01-1.08	NS
Dyslipidemia	0.46	0.25-0.80	0.006	0.42	0.21-0.83	0.013	-	-	-
Symptoms for >7 days	-	-	-	0.23	0.09-0.63	0.004	1.81	0.84-3.82	NS
Hydroxychloroquine	-	-	-	0.52	0.26-1.06	NS	10.53	2.23-49.69	0.003

Main outcome

In the univariate analysis of the composite outcome, age and active cancer were found to be significantly associated with 30-day mortality or need for ventilation while the presence of obesity and dyslipidemia were significantly associated with decreased odds. In the multivariable analysis, the adjusted effects of age and cancer remained significant, predicting that for each increase in a decade of age, the odds for the composite outcome increase 50%, and in the presence of active cancer, the odds for the composite outcome is 3.9 times higher. The presence of dyslipidemia was associated with a 55% decrease in the odds of 30-day mortality or ventilation.

Secondary outcomes

When 30-day mortality was evaluated individually, the univariate analysis found a significant association with higher age and the presence of active cancer and arterial hypertension. Patients with dyslipidemia, presenting with symptoms over a week or treated with hydroxychloroquine had significantly lower odds of being non-survivors. When adjusted for the significant variables in univariate analysis, for each decade of age the odds of 30-day mortality increased 100% and patients with active cancer had 6.8 times higher odds for 30-day mortality. Dyslipidemia and symptoms over one week were associated with 59% and 77% lower odds for 30-day mortality, respectively. The adjusted effect of the presence of arterial hypertension and the use of hydroxychloroquine was not significant in the final model.

In the univariate analysis considering the isolated 30-day need for ventilation, higher age and the presence of chronic kidney disease (CKD) and chronic cardiac disease were significantly associated with lower odds while symptoms duration over a week, chronic respiratory disease, and the use of hydroxychloroquine were significantly associated with higher odds for the need of ventilation. In the adjusted effects model, only chronic respiratory disease and the use of hydroxychloroquine remained significant and both were associated with increased odds for ventilation.

## Discussion

COVID-19 is a potentially severe disease that required worldwide health care and social readaptation. Local characteristics and risk factors for disease progression and worse outcomes in COVID-19 patients may vary between study populations, countries, and regions. Knowledge of these risk factors may help in the creation of algorithms for the identification of patients that benefit from closer follow-up, hospital admittance, and referral to intensive care. Our hospital is one of the reference centers in the north of Portugal and serves one of the largest populations in the country (approximately 700,000 people). When the study took place, this was the second county in the country with the highest number of COVID-19 cases (1658 confirmed cases), which is reflected in the high number of hospital admissions found in our cohort (271 admissions) during the first months of the pandemic. It is also important to highlight that, to our knowledge, this is the first Portuguese study analyzing the clinical characteristics of hospitalized COVID-19 patients and that all data were prospectively collected.

The clinical presentation of COVID-19 varies between mild and severe. While most infections present mildly, some patients progress rapidly to severe or critical stages, which are commonly associated with a hyperinflammatory state whose main manifestation is ARDS [6]. Studies suggest that dyspnea develops after a median of five to eight days since the beginning of the symptoms and hospital admission occurs after a median of seven days [7]. However, nearly a third may be asymptomatic [8]. Contrary to previous findings, our results suggest that patients admitted with prolonged symptoms are more likely to have a favorable outcome even after adjusting for strong predictors such as age. We could not identify a reason for this finding, but patients presenting to the hospital in the first week of symptoms may have had a more severe clinical presentation instead of a protracted or insidious development. Patients who are more debilitated are also less likely to be able to stay at home with a watchful waiting medical approach, which may justify our results.

**Figure 1:**
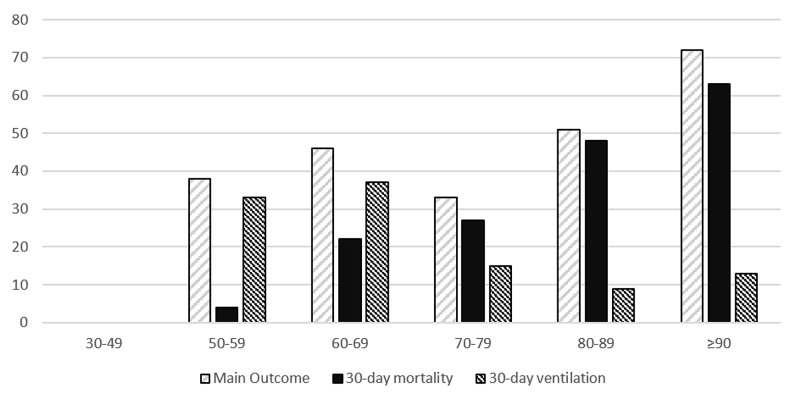
Outcomes per age class The X-axis represents age (years) and the Y-axis is the given percentage for the outcome. No measured events occurred in class 30-49 years.

In earlier studies, some clinical characteristics have been associated with the severity of COVID-19 [9-10] such as age, cancer, CKD, chronic obstructive pulmonary disease (COPD), immunocompromised state from solid organ transplant, obesity, heart failure, coronary heart disease, cardiomyopathies, sickle cell disease, or type 2 diabetes mellitus (T2D). In the present study, age and cancer, as expected, were also associated with higher mortality. Overall, mortality in our population was significantly higher than in most of COVID-19’s first wave of published studies: 35% vs 6.6% [11]. However, most studies described populations with mean ages ranging from 34 to 62 years old, which is significantly lower than the median age in our population (79 years old). This means we admitted an older population, which reflects a higher proportion of cardiovascular comorbidities and a higher likelihood of dying from COVID-19. As demonstrated in our results, mortality was almost null until the age of 60 but increased sharply after, reaching 63% in those ≥90 years old (Figure 1).

The frequency of T2D in patients with COVID-19 has been reported to range from 15% to 25% depending on disease severity and is one of the most frequently associated comorbidities in COVID-19 non-survivors [12], although no precise mechanism linking T2D with COVID-19 has been identified. Contrasting with previous findings, in our study, T2D did not significantly correlate with a COVID-19 prognosis, even though it was one of the most commonly reported comorbidities. One of the reasons behind this lack of association may be a selection bias, in which patients with multiple simultaneous cardiovascular risk factors may have been more easily admitted even though they presented with lesser severity due to a perceived risk of unfavorable outcomes. T2D is also known to be associated with other cardiovascular comorbidities, which themselves are believed to influence the prognosis of COVID-19 patients and, therefore, our multivariate analysis may have been underpowered. It was also not possible to quantify the degree of baseline glucose control and glycemic variability, which may have also influenced the prognosis.

Following initial concerns on a possible ACE-2 receptors upregulation by certain anti-hypertensive drugs, a meta-analysis suggested that the use of ACEI/ARB did not worsen the prognosis of COVID-19 and could even be protective in hypertensive subjects [13-15]. In our analysis, and as in previously published reports, arterial hypertension was associated with increased odds of dying, but this association did not persist after adjusting for age. We also could not find any association between ACEI/ARB use and mortality. Nevertheless, evidence suggests that ACEI/ARB therapy should not be discontinued in patients at risk for or who have COVID-19 [16].

A study from Shenzhen, China, identified obesity as a high-risk factor for developing severe pneumonia [17]. Furthermore, in another study from New York City, body mass index (BMI) >40 kg/m^2^ was the second strongest independent predictor of hospitalization, after age [18]. However, none of these studies found obesity as a risk factor for mortality in COVID-19 patients. Our findings suggest that obesity and hyperlipidemia may be protective in COVID-19 patients. However, we cannot find evidence supporting this as a true “obesity paradox” or just a result of an unquantified bias such as the presence of conditions associated with wasting such as smoking or prolonged disability in the elderly. As said before, our population was significantly older than what has been reported in most studies. In the elderly, obesity, opposed to cachexia, may signal a higher functional reserve. This association with dyslipidemia does not seem to be justified by the use of statins, as this variable was measured and had no association with the outcome.

During the first wave of the pandemic, hydroxychloroquine/chloroquine was used in many patients due to promising results in limited early non-randomized trials [19]. Lopinavir-ritonavir, a protease inhibitor, was also being used despite limited evidence of benefit [20-22]. Subsequent results from meta-analyses and randomized controlled trials failed to find a clinical benefit of either hydroxychloroquine/chloroquine [23] or lopinavir-ritonavir [24-25]. In agreement with the later studies, our results failed to find evidence of benefit in mortality with the use of these drugs to treat hospitalized COVID-19 patients. The use of these medications is no longer recommended outside randomized controlled trials. Other drugs, such as dexamethasone [26] and remdesivir [27-28], were, at the time, still on clinical trials and, unavailable or not, included in the treatment protocol and, therefore, were not evaluated.

The comorbidities and clinical features associated with the need for ventilation are even less described than for mortality. The need for ventilation has ranged from 20% to nearly 60% in patients with severe disease [29]. In our study, patients with a chronic pulmonary disease were more likely to be ventilated but the criteria used for intensive care admission were not always consistent. Patients with higher age (Figure 1) and multiple comorbidities were probably less likely to be ventilated, which probably contributed to a selection bias. There was also a significant correlation between the use of hydroxychloroquine and the need for ventilation that could also be due to a selection bias. Patients who were more fit for admission in an ICU, due to lower age and less cardiovascular comorbidities, would also be more prone to receive hydroxychloroquine. This is supported by the finding that in 27 patients that were admitted for comfort care (20 of them over 85 years old), only two received treatment with hydroxychloroquine.

Our population, older than the ones described in most studies during the first wave, might reflect better the COVID-19 incidence in Europe, where most countries have an increasingly older population structure [30]. This being an observational study, results regarding drug use should be seen with caution. As previously discussed, hydroxychloroquine/chloroquine and lopinavir-ritonavir use were nonrandomized and selection bias was probably present. Studies comparing mortality in earlier pandemic times with ongoing trials may provide a better understanding of mortality dynamics and identify risk populations in which interventions may be more effective.

## Conclusions

In our study, we found high mortality that is rarely seen in other infectious diseases. Age and cancer were the strongest predictors of mortality, whereas dyslipidemia was found to be protective. As seen in other scientific publications, hydroxychloroquine and lopinavir/ritonavir were not statistically associated with a better outcome. This data of first-wave patients will be useful to understand how our hospitalized patients and clinical approach have changed during the pandemic.

The increasing knowledge of COVID-19, the vaccination program, and the use of promising drugs may, hopefully, result in better treatment and improved outcomes, balancing the fears over new virus variants. It is essential to recognize that treating a recently discovered virus requires constant improvements in clinical practice using updated evidence-based medicine.
